# Draft genome sequence of *Amyloporia xantha* strain YAFMF0618, isolated from Gaoligong Mountain

**DOI:** 10.1128/mra.00240-24

**Published:** 2024-08-20

**Authors:** Xiaolei Zhao, Xiaolong Yuan, Yihang Yin, Jiaojun Yu, Yuan Zheng, Yi Wang

**Affiliations:** 1 College of Forestry, Southwest Forestry University, Kunming, China; 2 Yunnan Key Laboratory of Biodiversity of Gaoligong Mountain, Yunnan Academy of Forestry and Grassland, Kunming, China; 3 Hubei Key Laboratory of Economic Forest Germplasm Improvement and Resources Comprehensive Utilization, Huanggang Normal University, Huanggang, Hubei, China; University of Maryland School of Medicine, Baltimore, Maryland, USA

**Keywords:** *Amyloporia xantha*, genomes, nanopore sequencing

## Abstract

The *Amyloporia* genus is an important Chinese medicinal fungus. Here, we present a draft genome sequence of *Amyloporia xantha* strain YAFMF0618. The genome resource will support subsequent research into the potential secondary metabolite diversity of *A. xantha*.

## ANNOUNCEMENT

In recent decades, numerous pharmacological and phytochemical studies on fungus have proven that they are rich sources of novel compounds with various desirable activities (1). Polypore is a kind of fungi with abundant medicinal resources. This group of fungi produces a variety of secondary metabolites and has many biological activities such as antibacterial, anti-inflammatory, and antioxidant (2–4). *Amyloporia xantha* [Antrodia xantha (Fr.) Ryvarden] belongs to the family *Polyporaceae* (5), which has broad research prospects.

The *Amyloporia xantha* strain YAFMF0618 was collected from the bark of deciduous tree on Gaoligong Mountain, Yunnan province, China (25°17′ 34″ N, 99°16′ 47″ E) in 2020. After cleaning the collected sample, rinse it with running water for 48 h, then cut it into 2-cm pieces, and rinse it with distilled water. Then, soak in 70% ethanol for 2 min, rinse with 50 mL sterilized water six times, then soak in 10% sodium hypochlorite solution for 1 min, and rinse with 50 mL sterilized water 10 times. The 2-cm block was cut into tissue blocks of 0.5 cm in length and width and ground with a tissue grinder, and sterile water was added to obtain the slurry. Finally, the slurry was further diluted and spread on a solid malt yeast medium (Difco, Lawrence, KS, USA) containing 100 µg/mL ampicillin, calamycin, and streptomycin and incubated at 28°C for 20 days before individual colonies were transferred to the fresh solid malt yeast medium. After 30 days of culture in the medium, the strains were identified, and one colony (strain YAFMF0618) was identified as belonging to the genus Amyloporia by Internal Transcribed Spacer (ITS) region sequencing and the ITS sequence was deposited in GenBank (accession number: OR884097).

Genomic DNA was extracted using a Quant-iT PicoGreen dsDNA Assay Kit and used for both Illumina and Nanopore sequencing. Draft sequencing was performed using a whole-genome shotgun strategy (Personalbio technology Co. Ltd., Shanghai, China). The library preparation for the Illumina NovaSeq platform was performed using the TruSeq DNA Sample Prep Kit with insert sizes of 400 bp and a 2 × 150-bp paired-end configuration. High-quality sequences were obtained by trimming the adapter sequences with AdapterRemoval version 2 (6). And for the Nanopore library, preparation was executed using the ligation sequencing kit SQK-LSK109 (Oxford Nanopore Technologies, United Kingdom) according to the standard protocol provided by Oxford Nanopore Technologies. No shearing or size selection was performed during library preparation. The library was sequenced using a single FLO-MIN106 (R9.4.1) flow cell on a MinION sequencer. Guppy v6.1.5 was used for base calling, resulting in 817,533 reads with a N_50_ of 13,705 bp. Quality-filtered Illumina data and Nanopore data were used for *de novo* whole-genome assembly with Falcon and CANU v1.7.1 (7). Default parameters were used for all software unless otherwise specified. The assembly resulted in 60 scaffolds with a total assembly length of 46,808,119 bp (N_50_, 1,480,651 bp; N_90_, 321,224 bp) and a GC content of 51.54%(Table 1). The genome completeness was assessed using BUSCO v3.0.2 (8), showing a completeness score of 96.2%.

**TABLE 1:** Illumina sequencing, Nanopore sequencing, and genome assembly data

Parameter	Finding
Sample	*Amyloporia xantha* strain YAFMF0618
Illumina sequencing	
Total number of reads	40,379,776
Total bases (bp)	5,941,069,839
Nanopore sequencing	
Total sequence length (bp)	5,002,656,118
Total sequence number	817,533
Genome assembly	
Number of scaffold	60
Total length (bp)	46,808,119
N_50_ (bp)	1,480,651
N_90_ (bp)	321,224
GC content (%)	51.54
Complete BUSCOs (%)	96.2

## Data Availability

The draft genome sequence of *A. xantha* YAFMF0618 has been deposited at NCBI with accession number SAMN38610601 (GenBank accession number JAYKKR000000000), BioProject number PRJNA1048181, and SRA accession number SRR27452018.
